# Induction therapy prior to autologous stem cell transplantation (ASCT) in newly diagnosed multiple myeloma: an update

**DOI:** 10.1038/s41408-022-00645-1

**Published:** 2022-03-28

**Authors:** Abdul Hamid Bazarbachi, Rama Al Hamed, Florent Malard, Ali Bazarbachi, Jean-Luc Harousseau, Mohamad Mohty

**Affiliations:** 1 Department of Internal Medicine, Jacobi Medical Center, Albert Einstein College of Medicine, New York, USA; 2 Sorbonne University, Service d’Hematologie Clinique et Therapice Cellulaire, Hôpital Saint Antoine, and INSERM UMR 938, Paris, France; 3 Department of Internal Medicine, American University of Beirut, Beirut, 1107 2020 Lebanon; 4 Institut de Cancerologie de l’Ouest, Centre René Gauducheau, Nantes-St Herblain, France

**Keywords:** Myeloma, Targeted therapies

## Abstract

The current standard of care model for newly diagnosed fit multiple myeloma (NDMM) patients is the sequential treatment of induction, high dose melphalan, autologous stem cell transplantation (ASCT), and maintenance. Adequate induction is required to achieve good disease control and induce deep response rates while minimizing toxicity as a bridge to transplant. Doublet induction regimens have greatly fallen out of favor, with current international guidelines favoring triplet or quadruplet induction regimens built around the backbone of the proteasome inhibitor bortezomib and dexamethasone (Vd). In fact, the updated 2021 European Haematology Association (EHA) and European Society for Medical Oncology (ESMO) clinical practice guidelines recommend the use of either lenalidomide-Vd (VRd), or daratumumab-thalidomide-Vd (Dara-VTd) as first-line options for transplant-eligible NDMM patients, and when not available, thalidomide-Vd (VTd) or cyclophosphamide-Vd (VCd) as acceptable alternatives. Quadruplet regimens featuring anti-CD38 monoclonal antibodies are extremely promising and remain heavily investigated, as is the incorporation of more recent proteasome inhibitors such as carfilzomib. This review will focus on induction therapies prior to ASCT examining the latest data and guidelines on triplet and quadruplet regimens.

## Introduction

Thirty-five years after the introduction of high dose melphalan (HDM) followed by autologous stem cell transplantation (ASCT) as a treatment strategy for multiple myeloma (MM) [1–3], ASCT remains the standard of care for newly diagnosed eligible patients. This strategy phases are fourfold, induction therapy, HDM, ASCT+/− consolidation, and maintenance, a model associated with high response rates, prolonged progression-free survival (PFS), and overall survival (OS).

The ultimate goal behind induction therapy is to achieve adequate disease control and induce the deepest possible response with emerging data suggesting an added benefit from achieving minimal residual disease (MRD) negativity, all while minimizing toxicity and allowing adequate stem-cell harvest as a bridge to transplant.

The previously popular doublet induction regimens have greatly fallen out of favor, and current international guidelines favor triplet or quadruplet induction regimens. In 2017, the European Society for Medical Oncology (ESMO) proposed that for patients aged <65 (or fit older patients up to 70 years) eligible for ASCT, a systemic triplet induction be used comprising the proteasome inhibitor (PI) bortezomib plus dexamethasone (Vd) backbone, with the addition of a third agent such as thalidomide (VTd), cyclophosphamide (VCd), lenalidomide (VRd) or doxorubicin (PAd) [4]. This would be followed by the standard preparative regimen of intravenous melphalan 200 mg/m^2^ and ASCT with the immunomodulatory imide drug (IMiD) lenalidomide maintenance. The updated 2021 European Haematology Association (EHA) and ESMO guidelines however incorporated more novel agents and provided simpler frontline strategy outside clinical trials, recommending VRd or VTd plus the CD38 monoclonal antibody (mAb) daratumumab (Dara-VTd) as first options, and when unavailable, VTd or VCd as second options, followed by ASCT and lenalidomide maintenance [5].

This review will focus on induction therapies prior to ASCT highlighting the latest data on triplet and quadruplet regimens.

## ASCT as a standard of care

As mentioned earlier, frontline ASCT remains the current standard of care, based on three major trials: IFM/DFCI2009, EMN02/HO95, and FORTE (Table 1).

**Table 1:** ASCT landmark trials.

Study	No.	Treatment			Median follow-up	Outcome			
		Induction	Consolidation	Maintenance					
IFM/DFCI2009	350	3 cycles VRd	ASCT + 2 cycles VRd	1 year lenalidomide	93 months	**Median PFS**	**Adjusted HR**	**OS rate**	**Adjusted HR**
						47.3 months	0.70 (*p* = 0.0001)	62.20%	1.03 (*p* = 0.815)
	350		5 cycles VRd			35 months		60.20%	
EMN02/HO95	702	ASCT (single or tandem)	Randomized 2 cycles VRd vs no consolidation	Lenalidomide until progression	60.3 months	**Median PFS**	**Adjusted HR**	**OS rate (75 months)**	**Adjusted HR**
						56.7 months	0.73 (*p* = 0.0001)	69%	0.80 (*p* = 0.0342)
	495	4 cycles VMP				41.9 months		63%	
FORTE	157	4 cycles KRd	ASCT + 4 cycles KRd	Randomized KR vs lenalidomide alone until progression	45 months	**3-year PFS**		**Adjusted HR**	
						78%		0.64 (*p* = 0.023) vs KRd	0.53 (*p* < 0.001) vs KCd-ASCT
	158	12 cycles KRd				68%		0.82 (*p* = 0.262) KRd vs KCd-ASCT	
	159	4 cycles KCd	ASCT + 4 cycles KCd			58%			

*VRd* velcade, revlimid, dexamethasone, *ASCT* autologous stem-cell transplantation, *PFS* progression-free survival, *HR* hazard ratio, *OS* overall survival, *VMP* velcade, melphalan, prednisone, *KRd* carfilzomib, lenalidomide, dexamethasone, *KCd* carfilzomib, cyclophosphamide, dexamethasone.

### IFM/DFCI2009

The IFM/DFCI2009 trial is a multicenter, randomized, open-label phase 3 study that included 700 newly diagnosed MM (NDMM) patients aged <66 years, randomly assigned to receive induction therapy with three cycles of VRd and then consolidation therapy with either five additional cycles of VRd (350 patients) or ASCT followed by two additional cycles of VRd (350 patients) [6]. Patients in both groups received maintenance therapy with lenalidomide for 1 year. The primary endpoint was PFS. Median PFS was significantly longer in the transplant group compared to the VRd-only group (50 months versus vs 36 months; adjusted hazard ratio [HR] 0.65; *p* < 0.001). Even after long-term follow-up (median 93 months), nearly identical results were reported at the American Society of Haematology (ASH) meeting in 2020 with a median PFS of 47.3 months for ASCT vs 35 months for VRd alone, equivalent to a 30% reduction in the risk of progression or death (HR 0.70; *p* = 0.0001) [7]. However, after 8 years follow-up, no significant difference in OS was noted between the two groups, with rates of 62.2% vs 60.2% for transplant and VRd alone, respectively (HR 1.03; *p* = 0.815) [7]. It is important to note that by design, patients who were randomized to the VRd-only group underwent ASCT at disease progression as salvage treatment, and with longer follow-up, a big proportion of patients had already underwent salvage transplantation. These results imply that early vs late ASCT have similar outcomes, and that patients who receive frontline VRd alone can still be salvaged with ASCT at the time of relapse. The transplant arm did however achieve higher rates of MRD-negativity (30%) assessed by next generation sequencing (NGS) at a sensitivity of 10^−6^ compared to VRd alone (20%). Interestingly, patients achieving MRD-negativity appear to have an OS benefit both before and after maintenance, whereby MRD-negative patients at the start of maintenance therapy had 94% 4-years OS compared to 79% in MRD-positive patients (HR 0.24; *p* = 0.001), and similarly, MRD-negative patients after 12 months of maintenance had 96% 3-years OS (after maintenance completion) compared to 86% in MRD-positive patients (HR 0.26; *p* = 0.008). MRD-negativity appears to be a possible surrogate for OS and could be used as an important endpoint for future consideration, regardless of risk profile or disease stage [8, 9].

### EMN02/HO95

The second study to support the use of ASCT as intensification therapy was the EMN02/HO95, a multicenter, randomized, open-label, phase 3 trial that included 1503 NDMM patients [10]. It comprised two randomization stages, firstly to intensification therapy with either upfront ASCT or four 42-day cycles of bortezomib-melphalan-prednisone (VMP), and secondly to receive two 28-day VRd consolidation cycles or no consolidation, with all groups receiving lenalidomide maintenance therapy. In centers with a double HSCT policy, the first randomization was to VMP, or single or double HSCT. The rate of very good partial response or better (≥VGPR) was 84% in the ASCT group vs 77% in the VMP group (*p* = 0.0021), and after a median follow-up of 60.3 months, there was a benefit of almost 15 months in terms of median PFS with 56.7 vs 41.9 months for frontline ASCT vs VMP, respectively (HR 0.73; *p* = 0.0001). No OS difference was found between these groups. After an extended follow-up of 75 months, however, there appeared to be a slight OS advantage in favor of ASCT vs VMP, with rates of 69% vs 63%, respectively (HR 0.80; *p* = 0.03) [11]. The study concluded that upfront ASCT significantly prolonged OS compared to VMP alone.

### FORTE

The third study to support frontline HDT as the standard of care is the FORTE trial, a multicenter, randomized, open-label, phase 3 trial sponsored by the European Myeloma Network, which comprised 474 NDMM transplant-eligible patients aged ≤65 years [12]. It compared carfilzomib, lenalidomide, and dexamethasone (KRd) with (158 patients) or without ASCT (157 patients), with carfilzomib, cyclophosphamide, and dexamethasone (KCd) plus ASCT (159 patients). After a median follow-up of 50.9 months from the first randomization (induction/consolidation treatment), 4-year PFS rates for KRd-ASCT, KRd alone, and KCd-ASCT were 69%, 56%, and 51%, respectively. The observed advantage for KRd-ASCT was in fact significant, resulting in better PFS compared to KCd-ASCT (HR 0.54; *p* = 0.0008) and KRd alone (HR 0.61; *p* = 0.008). No significant difference was noted between KRd and KCd-ASCT (HR 0.82; *p* = 0.3). The rate of 1-year sustained MRD negativity was higher in the KRd-ASCT group (47%) than in the KRd alone (35%).

Compared to modern non-intensive therapies, ASCT improves the response rate and the incidence of MRD negativity, which translates into an increased median PFS and a higher percentage of patients achieving long-term PFS in all prognostic subgroups including high-risk cytogenetics. This explains why ASCT remains the standard of care for all fit eligible patients and is mandatory in high-risk patients. It should be noted however that the OS benefit, if it exists, is delayed because of active salvage treatments.

## Triplet induction regimens prior to ASCT

The superiority of triplet-based regimens over doublet-based strategies have long been established [13–15]. As previously discussed, the 2017 ESMO guidelines [4] recommend the use of either VRd, VTd, VCd, or PAd, the latter being largely considered outdated (Table 2).

**Table 2:** Triplet induction regimens prior to ASCT.

Study	No.	Treatment		Outcome			
		Induction	Consolidation				
IFM2013-04	169	4 cycles VTd	ASCT (single or tandem ± consolidation and/or maintenance at center direction)	**VGPR rate**		**ORR rate**	
				66.30%		92.30%	
	169	4 cycles VCd		56.20%		83.40%	
				*p* = 0.05		*p* = 0.01	
PETHEMA/GEM2012	458	6 cycles VRd	ASCT + 2 cycles VRd	**CR post induction**	**CR post ASCT**		**CR post consolidation**
				33.40%	44.10%		50%
Integrated analysis (PETHEMA/GEM2012; PETHEMA/GEM2005)	407	6 cycles VRd	ASCT	**VGPR post induction**	**VGPR post ASCT**	**MRD-post-induction**	**MRD-post-ASCT**
				66.30%	74.40%	46.70%	62%
	129	6 cycles VTd		51.20%	53.50%	34.90%	47.30%
				OR (95% CI) 1.87 (1.23–2.83)	OR (95% CI) 2.52 (1.64–3.87)	OR (95% CI) 1.39 (0.87–2.22)	OR (95% CI) 1.7 (0.94–3.05)
Integrated analysis (IFM2009; IFM2013-04)	331	3 cycles VRd	ASCT	**VGPR post induction**			
				57.10%			
	154	4 cycles VTd		56.50%			
				OR (95% CI) 1.06 (0.71-1.59)			
FORTE	187	4 cycles KRd	ASCT + 4 cycles KRd or 8 cycles KRd	**VGPR post induction**			
				74%			
	94	4 cycles KCd	ASCT + 4 cycles KCd	61%			
				*p* = 0.05			

*VTd* velcade, thalidomide, dexamethasone, *VCd* velcade, cyclophosphamide, dexamethasone, *VRd* velcade, revlimid, dexamethasone, *KRd* carfilzomib, lenalidomide, dexamethasone, *KCd* carfilzomib, cyclophosphamide, dexamethasone, *ASCT* autologous stem-cell transplantation, *VGPR* very good partial response, *ORR* overall response rate, *CR* complete response, *MRD* minimal residual disease, *OR* odds ratio, *CI* confidence interval.

### VTd vs VCd

In 2016, a prospective trial (IFM2013-04) compared induction with four cycles of VTd vs four cycles of VCd in 338 patients and found significantly higher response rates with VTd using IMWG criteria with VGPR rates of 66.3% and 56.2%, respectively (*p* = 0.05), and overall response rates (ORR) of 92.3% vs 83.4% (*p* = 0.01) [16]. VCd however remains as a therapeutic option despite its inferiority, mainly as a cost-effective regimen when resources are limited, or when specific patient comorbidities such as renal failure and severe neuropathy limit the usage of other drugs such as IMiDs.

### VRd

The Spanish PETHEMA/GEM2012 trial published in 2019 recruited 458 patients aged ≤65 years who received six cycles of VRd induction followed by ASCT and two additional post-transplant consolidation cycles [17]. Responses were grouped by induction, transplant, and consolidation, revealing a continuous stepwise deepening of response. In the 426 patients who completed six cycles of induction, the rates of ≥VGPR were 55.6% by cycle 3, 63.8% by cycle 4, 68.3% by cycle 5, and 70.4% after induction completion. In the intent-to-treat (ITT) population, the complete response (CR) rate after induction was 33.4%, 44.1% after ASCT and 50.2% after consolidation. MRD data from the same study in 317 patients using NGS revealed a similar pattern of stepwise improvement, with MRD-negativity rates of 35%, 54%, and 58%, after six cycles of induction, ASCT, and consolidation, respectively [18]. This study showed VRd to be an effective and well-tolerated regimen for induction with deepening response throughout induction and over the course of treatment.

### VRd vs VTd

While there are no randomized controlled trials to date directly comparing VTd and VRd, the Spanish Myeloma Group (PETHEMA/GEM) performed two consecutive trials on transplant-eligible NDMM patients, one published in 2012 looking at six cycles of VTd [19] and the other using six cycles of VRd [17]. Data from these two trials were integrated and analyzed, and both the ≥VGPR (post-induction and post-ASCT) and the MRD-negativity rates (10^−4^ sensitivity) were better with VRd than with VTd, translating into a better 1-year (89.2% vs 83%) and 2-year PFS (81.5% vs 69.0%) favoring VRd [20]. This integrated analysis met its primary endpoint of noninferiority, and demonstrated a statistically significant and clinically relevant improvement of the ≥VGPR rate after induction with VRd vs VTd (66.3% vs 51.2%; *p* = 0.003), ≥VGPR rate post-ASCT (74.4% vs 53.5%), MRD-negativity rate post-induction (46.7% vs 34.9%), and MRD-negativity post-ASCT (62.4% vs 47.3%). Furthermore, VTd was associated with higher rates of peripheral neuropathy, with grade 2, 3, and 4 toxicity of 46%, 12%, and 2%, respectively [19], compared to 13%, 1%, and 0%, respectively with VRd [17]. Therefore, it can be potentially concluded that VRd is both more efficacious and less toxic than VTd.

### KRd vs KCd

With this background, the triplet combination of the second-generation PI carfilzomib plus lenalidomide and dexamethasone (KRd) could prove to be attractive. The FORTE study discussed above looked at the survival of transplant-eligible patients after randomization to KRd vs KCd [12]. After four induction cycles, the ≥VGPR rate was around 74% for KRd vs 61% for KCd [12]. Despite a good safety profile and inducing a deep response, KRd is not yet approved by the European Commission and is not used outside clinical trials.

### VRd vs KRd

No trial to date compared the current standard VRd to KRd as pre-transplant induction, but data can be extrapolated from the phase 3 ENDURANCE trial evaluating the two combinations in NDMM patients not considered for immediate ASCT [21]. The trial included 1087 patients randomized to either KRd (545 patients) or VRd (542 patients), and at a median follow-up of 9 months, no significant difference in PFS was observed between the two groups (HR 1.04; *p* = 0.74). KRd however leads to more toxicity, making VRd a better backbone for the treatment of NDMM.

In summary, the triplet regimen VRd appears to be superior to VTd, which in turn is better than VCd, and is currently the preferred triplet-based induction therapy for NDMM.

## Quadruplet induction regimens prior to ASCT

Building on the triplet foundation, the next step would be to assess whether a quadruplet-based induction regimen could further increase response rates and improve outcomes (Table 3).

**Table 3:** Quadruplet induction regimens prior to ASCT.

Study	No.			Treatment		Median follow-up	Outcome					
				Induction	Consolidation							
CASSIOPEIA	543			4 cycles Dara-VTd	ASCT + 2 cycles Dara-VTd	18.8 months	**sCR (day 100 post ASCT)**	**≥CR**	**≥VGPR**	**PFS rate**	**OS rate**	**MRD-negativity (10-5)**
							29%	39%	83%	93%	97%	64%
	542			4 cycles VTd	ASCT + 2 cycles VTd		20%	26%	78%	85%	93%	44%
							OR 1.60 (*p* = 0.001)	*p* < 0.0001	*p* = 0.024	HR 0.47 (*p* < 0.0001)	HR (95% CI) 0.43 (0.23–0.80)	*p* < 0.0001
Matching-adjusted indirect comparison (CASSIOPEIA, IFM2009, GMMG-MM5, IFM2005-01)	529 matched to VRd	206 matched to VCd	416 matched to Vd	Dara-VTd	ASCT	–	**PFS control**			**OS control**		
	350			VRd			HR (95% CI) 0.47 (CI 0.33–0.69)			HR (95% CI) 0.31 (0.16–0.57)		
	126			VCd			HR (95% CI) 0.35 (0.21–0.58)			HR (95% CI) 0.35 (0.14–0.86)		
	240			Vd			HR (95% CI) 0.42 (0.28–0.63)			HR (95% CI) 0.38 (0.18–0.77)		
GRIFFIN	104			4 cycles Dara-VRd	ASCT + 2 cycles Dara-VRd	38.6 months	**sCR**	**MRD-negativity (10-5)**	**MRD-negativity (10-6)**	**Sustained MRD-negativity (10-5) ≥12 months**	**Median PFS**	**3-year PFS**
							66%	64%	35.6%	44%	Not reached	88.9%
	103			4 cycles VRd	ASCT + 2 cycles VRd		47%	30%	14.6%	13%	Not reached	81.2%
							*p* = 0.0096	*p* < 0.0001	*p* = 0.0007	*p* < 0.0001	HR 0.46; 95% CI, 0.21–1.01	–
MASTER	123			4 cycles Dara-KRd	ASCT + 0, 4 or 8 cycles Dara-KRd (MRD driven)	–	**≥CR**	**MRD negativity (10-5)**	**MRD negativity (10-6)**	**2-year PFS**	**2-year OS**	
							86%	80%	66%	87%	94%	
GMMG-HD7	331			3 cycles Isa-VRd	ASCT	–	**≥VGPR**			**MRD-negativity (10-5)**		
							77.30%			50.10%		
	329			3 cycles VRd			60.50%			35.60%		
							*p* < 0.001			OR 1.83, *p* < 0.001		
GMMG-CONCEPT	50			6 cycles Isa-KRd	ASCT + 4 cycles Isa-KRd	–	**MRD-post induction**					
							60%					
IFM 2018-01	45			6 cycles Dara-IRd	ASCT + 4 cycles Dara-IRd	23.6 months	**≥VGPR**	**MRD-negativity (10-5)**		**MRD-negativity (10-6)**	**2-year PFS**	
							93.4%	51.4%		39.5%	95.2%	
GMMG-HD6	279			4 cycles Elo-VRd	ASCT + 2 cycles Elo-VRd	49.8 months	**≥VGPR**		**3-year PFS**		**3-year OS rate**	
							78.9% (R maintenance); 78.2% (Elo-R maintenance)		68.8% (R maintenance); 68.5% (Elo-R maintenance)		89.4% (R maintenance); 89.1% (Elo-R maintenance)	
	280			4 cycles VRd	ASCT + 2 cycles VRd		81.5% (R maintenance); 80.7% (Elo-R maintenance)		66.2% (R maintenance); 67.2% (Elo-R maintenance)		92.5% (R maintenance); 89.7% (Elo-R maintenance)	
							*p* = 0.95		*p* = 0.86		*p* = 0.43	

*Dara* daratumumab, *VTd* velcade, thalidomide, dexamethasone, *VRd* velcade, revlimid, dexamethasone, *VCd* velcade, cyclophosphamide, dexamethasone, *KRd* carfilzomib, lenalidomide, dexamethasone, *Ixa* isatuximab, *IRd* ixasomib, lenalidomide, dexamethasone, *elo* elotuzumab, *ASCT* autologous stem-cell transplantation, *sCR* stringent complete response, *VGPR* very good partial response, *PFS* progression-free survival, *OS* overall survival, *MRD* minimal residual disease, *OR* odds ratio, *HR* hazard ratio, *CI* confidence interval.

### Dara-VTd

The CASSIOPEIA phase 3 study included 1085 NDMM patients aged 18–65 years and evaluated whether the addition of daratumumab to VTd (Dara-VTd) before and after ASCT would improve outcomes [22]. In Part 1 of this study (Induction and Consolidation), patients were randomized 1:1 to receive either four pre-transplant induction and two post-transplant consolidation cycles of VTd alone or in combination with daratumumab. The primary endpoint was stringent CR (sCR). Almost all patients (Dara-VTd = 90%, VTd = 89%) were able to proceed to ASCT demonstrating the feasibility of this regimen in routine practice. Dara-VTd increased depth of response, translating into improved PFS with an acceptable safety profile. In fact, before ASCT, the ≥VGPR rate was 65% and 56% in the Dara-VTd and VTd groups, respectively, and the rates post-ASCT were 76% and 67%, respectively. At day +100 post-transplant, in the ITT population, 29% of patients in the Dara-VTd group and 20% in the VTd group had achieved the primary endpoint of a sCR (odds ratio [OR] 1.60; *p* = 0.001). Furthermore, 39% of patients in the Dara-VTd group vs 26% in the control group achieved a ≥CR. This depth of response translated into a better PFS rate of 93% vs 85% in favor of the Dara-VTd group, and while the median PFS from first randomization was not reached in either arm, there was a 53% reduction in the risk of progression or death in the Dara-VTd group (HR 0.47; *p* < 0.0001) [22]. Although OS data are still immature after a median follow-up of only 18.8 months, there seems to be a trend towards better OS with rates of 97% vs 93% for Dara-VTd vs VTd, respectively (HR 0.43, 95% CI 0.23-0.80) [22]. A longer follow-up is needed to confirm this benefit. The CASSIOPEIA study showed that Dara-VTd was superior across all subgroups (including high-risk cytogenetics, and International Staging System [ISS] Stage III disease) and was the first study to show the clinical benefit of adding daratumumab to standard of care in transplant-eligible patients. Based on these data, Dara-VTd was approved by the European Commission in January 2020, the first approved regimen in over six years for transplant-eligible NDMM patients.

Following both induction and consolidation, the rates of MRD-negativity were significantly higher in the Dara-VTd group (9.2% vs 5.4%; OR 1.79; *p* = 0.02), and (33.7% vs 19.9%; OR 2.06; *p* < 0.0001), respectively [23]. Sustained MRD-negativity rates were also higher in the Dara-VTd group at 1-year (50.1% vs 30.1%; OR 2.37; *p* < 0.0001) and at 2 years (35.5% vs 18.8%; OR 2.41; *p* < 0.0001). Achieving MRD-negativity was associated with improved PFS in both treatment groups, whereby patients with 1-year and 2-years sustained MRD-negativity had HR of 0.20 (*p* < 0.0001), and 0.08 (*p* < 0.0001), respectively. This was also noted in the Data-VTd group specifically, with 1-year and 2-years sustained MRD-negativity associated with HR of 0.20 (*p* < 0.0001) and 0.04 (*p* < 0.0001), respectively. The use of daratumumab maintenance compared to observation was also independently associated with significantly increased MRD negativity rates (58.6% vs 47.1%; OR 1.80; *p* = 0.0001); however, it is worth noting that in contrast to patients who had received VTd only induction/consolidation, no significant advantage was noted in the Dara-VTd group, and the rates of MRD-negativity and sustained negativity at 1 and 2 years were similar between daratumumab maintenance and observation alone. This implies that the use of daratumumab as maintenance therapy is only advantageous in daratumumab naive patients, and that its use during induction and consolidation is likely enough.

A direct comparison between Dara-VTd and VRd does not currently exist, but a matching-adjusted indirect comparison (MAIC) of PFS and OS has been undertaken using data from CASSIOPEIA and other trials evaluating VRd, VCd and Vd. After matching adjustment, significant improvements in PFS were estimated for Dara-VTd vs VRd (HR 0.47, 95% CI 0.33–0.69), VCd (HR 0.35, 95% CI 0.21–0.58) and Vd (HR 0.42, 95% CI 0.28–0.63) [24]. Results for OS were also better for Dara-VTd vs VRd (HR 0.31, 95% CI 0.16–0.57), VCd (HR 0.35, 95% CI 0.14–0.86) and Vd (HR 0.38, 95% CI 0.18–0.77). This analysis suggests that Dara-VTd is the best combination for transplant-eligible NDMM, and given these findings, the next step would be to investigate the Dara-VRd combination.

### Dara-VRd

The phase 2 GRIFFIN trial randomized 207 transplant-eligible NDMM patients to receive either Dara-VRd (104 patients) or VRd (103 patients) alone [25]. The addition of daratumumab was associated with increased rate of sCR after prolonged follow-up (median 27.4 months) 63.6% vs 47.4% in the control group (*p* = 0.03), as well as MRD-negativity [10^−5^] rate (62.5% vs 27.2%, *p* < 0.0001). Updated results were presented at ASH 2021 after 24 months of maintenance therapy (DR vs R, median follow-up 38.6 months) [26], and the rates of sCR significantly favored the Dara-VRd group in the response-evaluable population at 66% vs 47.4% (*p* = 0.0096), as did rates of MRD-negativity both at 10^−5^ (64.4% vs 30.1%; *p* < 0.0001) and 10^−6^ (35.6% vs 14.6%, *p* = 0.0007) with sustained MRD-negativity [10^−5^] lasting ≥12 months of 44.2% in the Dara-VRd group vs 12.6% in VRd alone (*p* < 0.0001). Median PFS was not reached in either arm after 38.6 months follow-up but did favor the daratumumab group (HR 0.46; 95% CI, 0.21–1.01), with rates of 36-months PFS of 88.9% and 81.2% for Dara-VRd and VRd, respectively.

The ongoing phase 3 PERSEUS international registration study (NCT03710603) is currently evaluating subcutaneous daratumumab in combination with VRd vs VRd in 690 European transplant-eligible patients with NDMM [27]. The results of this study are likely needed before opting for subcutaneous formulations of daratumumab as the treatment of choice.

### Dara-KRd

As previously mentioned, the second-generation PI carfilzomib is more potent than its predecessor bortezomib, and its use in lieu of bortezomib in combination with daratumumab could prove highly effective.

The MASTER trial is currently evaluating upfront Dara-KRd in NDMM, whereby patients receive four cycles of induction, followed by ASCT, and either 0, 4 or 8 additional cycles of Dara-KRd consolidation according to MRD status until achieving two consecutive negative reads (<10^−5^), either post-induction and post-transplant, or post-transplant and during consolidation [28]. The study accrued 123 patients, 20% of which were 70 or older, over a median follow-up of 25.1 months. Response ≥CR was obtained in 86% of patients, and 80% and 66% of patients have achieved MRD negativity <10^−5^ and <10^−6^, respectively. Depth of response had stepwise improvement with each additional phase of therapy, and importantly, became similar across risk groups including patients with 0, 1, and 2+ high-risk cytogenetic abnormalities (78%, 82%, and 79%, respectively) using MRD-guided consolidation. Two-year PFS was 87%, and 2-year OS 94%. This is the first report of a mAb-based quadruplet regimen with MRD response-adapted therapy in NDMM. The combination appears safe and effective and can possibly overcome high-risk diseases.

### Dara-IRd

Replacing bortezomib with ixazomib in combination with lenalidomide and dexamethasone (IRd) as an all-oral regimen has already been demonstrated to be safe, convenient, and effective [29], which naturally led to investigate the addition of daratumumab to this triplet combination.

The phase 2 IFM 2018-01 study investigated this combination, and enrolled 45 patients to receive six cycles of Dara-IRd induction followed by ASCT and four additional Dara-IRd consolidation cycles with 2 years lenalidomide maintenance [30]. Primary endpoint was MRD-negativity post consolidation [10^−6^]. All patients responded to treatment, with 93.4% achieving ≥VGPR. MRD-negativity rates in the 38 evaluable patients were 39.5% and 51.4% at 10^−6^ and 10^−5^, respectively. The 2-year PFS rate was 95.2%, whereby after a median follow-up of 23.6 months, only two patients experienced disease progression. Dara-IRd appears to be safe and effective, however, it is worth noting that MRD-negativity rates using this regimen were lower than those obtained with more mainstream regimens such as Dara-VTd, Dara-VRd, or Dara-KRd. More follow-up is needed to determine the long-term outcomes of this combination.

### Isa-VRd

In light of daratumumab’s success, the anti-CD38 mAb isatuximab is also being investigated as part of a quadruplet-based induction. The phase 3 GMMG-HD7 trial included 660 patients that were randomized to either VRd (329 patients) or Isa-VRd (331 patients) [31]. Response rates were significantly higher in the isatuximab group with ≥VGPR rates of 77.3% vs 60.5% in the control group (*p* < 0.001), as were rates of MRD-negativity of 50.1% vs 35.6% in the control group (OR = 1.83, *p* < 0.001). Isatuximab was not associated with increased rates of serious adverse events, and the combination was deemed safe and effective.

### Isa-KRd

Isatuximab is also being evaluated in the GMMG-CONCEPT trial, in combination with KRd in cytogenetically high-risk young (Arm A ≤ 70 years) and elderly (Arm B > 70 years) NDMM patients [32, 33]. Six induction cycles of Isa-KRd are given before ASCT (or two additional induction cycles in transplant ineligible patients) followed by four consolidation cycles and Isa-KR maintenance. Fifty patients have so far been included in the study (46 arm A, 4 arm B), all of which responded to treatment with at least PR, and 90% ≥VGPR. Median PFS was not reached after a median follow-up of 24.9 months, with 2-year PFS rate of 75.5%. Of 33 patients tested (31 evaluable) for MRD after induction, 20 patients (65%) were MRD negative. In this first-time trial investigating Isa-KRd quadruplet solely in high-risk NDMM, the combination was shown safe and effective in inducing deep responses in this challenging group. The study continues to recruit patients.

### Elo-VRd

Most quadruplet regimens currently under investigation incorporate a CD38-directed mAb to the PI/IMiD/dex backbone, however, alternatives are also being explored. The anti-SLAMF7 mAb elotuzumab has proven quite effective in the relapsed/refractory setting in the ELOQUENT-2/-3 trials [34–36], and is being investigated in combination with VRd in NDMM.

The phase 3 GMMG-HD6 trial is the first to evaluate elotuzumab in transplant eligible NDMM, and analyzed 559 patients randomized to receive either four induction cycles and two post-ASCT consolidation cycles with either Elo-VRd (279 patients) or VRd alone (280 patients), followed by a second randomization to either lenalidomide or elotuzumab-lenalidomide maintenance for 2 years [37]. No differences in rates of ≥VGPR were noted across all four groups which ranged from 78.9 to 81.5%, and despite a median follow-up of 49.8 months, PFS and OS were also similar. The addition of elotuzumab to VRd in the upfront setting therefore does not appear to have any notable advantage, which is also in line with previous reports from the ELOQUENT-1 and SWOG-1211 trials, and its use should be reserved for the relapsed/refractory setting only.

## Optimal number of cycles and the role of consolidation

The number of induction cycles in the aforementioned studies varied from 3 to 6, and no randomized trial specifically addressed this question. It is worth noting that in the PETHEMA/GEM2012 trial, the rate of ≥VGPR had a stepwise increase with increasing number of cycles, from 55.6% by cycle 3 to 70.4% by cycle 6, and that this post-induction ≥VGPR rate was apparently better than in the IFM/DFCI2009 trial where only three cycles of VRd were used for induction.

The number of cycles can also impact post-transplant consolidation, especially since the same induction regimen is usually used (except in the EMN02 trial). Indeed, the benefit of an additional two cycles of consolidation was higher in the IFM/DFCI2009 trial since the post-consolidation ≥VGPR rate increased to 78% compared to the PETHEMA/GEM2012 trial where it only increased to 75.5%. It appears that the influence of consolidation greatly depends on the results of induction, and that the real impact stems from the total number of cycles used between induction and consolidation, which should probably consist of at least 8 as was the case in the PETHEMA/GEM2012 (6 + 2) and the FORTE (4 + 4) trials.

## Safety of induction regimens prior to ASCT

A major consideration when choosing an appropriate induction regimen is potential associated toxicity centered around drug-specific reactions and patient comorbidities. In the major studies discussed above, treatment-related death rates were extremely low, ranging from 0% (0/536) with four cycles of Dara-VTd and 0.7% (4/538) with four cycles of VTd in the CASSIOPEIA trial, 0% (0/104) with four cycles of Dara-VRd and 1% (1/103) with four cycles of VRd in the GRIFFIN trial, to 1.1% (5/458) with six cycles of VRd in the PETHEMA GEM2012 trial and 1.3% (20/1493) with four cycles of VCd in the EMN02/HO95 trial. The most common grade III–IV adverse events encountered with these regimens, as expected, were myelosuppression and peripheral neuropathy. Rates of severe neutropenia ranged from 15 to 22% with VTd and VRd, up to 28 and 41% with the addition of daratumumab (Dara-VTd & Dara-VRd, respectively) [22, 25]. Similarly, grade III–IV thrombocytopenia were reported in 7–9% with the use of VTd and VRd, and up to 11 and 16% with the addition of daratumumab (Dara-VTd & Dara-VRd, respectively) [22, 25]. Rates of grade III–IV peripheral neuropathy were comparable between all four major regimens around 7–9% [22, 25]. Daratumumab was also associated with infusion reactions in around 40% of patients, 10% of which graded III–IV [22, 25]. It was also evident that with the increased use of immunomodulatory drugs (IMiDs) and anti-CD38 mAbs, the median stem-cell harvest has significantly decreased (range 6.0–10.58 × 10^6^ CD34+/kg) necessitating increased usage of the hematopoietic stem-cell mobilizer plerixafor to achieve an adequate harvest [38–43]. Despite the lower yield and higher plerixafor use with the addition of daratumumab however, there doesn’t seem to be an impact on the feasibility and safety of ASCT, and overall successful engraftment post-transplant was not hindered [40].

## Concluding remarks

Induction therapy is the most crucial step in myeloma treatment, and the goal behind it should be achieving the deepest possible response which directly correlates with long-term outcomes. Triplet-based therapies featuring a PI, IMiD, and dexamethasone remain the current standard of care, but recent data has shown that a quadruplet approach incorporating anti-CD38 mAbs could have a superior outcome. The recently updated 2021 EHA-ESMO clinical practice guidelines recommend the use of either VRd or Dara-VTd as first-line options for transplant-eligible NDMM patients—the latter being the more optimal choice—and when not available, VTd or VCd as acceptable alternatives. Attempts at replacing bortezomib with newer PI at the frontline were met with increased toxicity and lack of outcome improvement, and while lenalidomide has proven to be the best current option, we are yet to see newer generation IMiDs such as pomalidomide being investigated in this setting as part of induction. It remains unclear how many induction cycles should be used, and while higher numbers are associated with deeper responses, they also carry worse adverse events [44]. A response-adapted approach as seen in the MASTER trial could prove promising, and possibly eliminate the notion of one size fits all. It remains a matter of debate whether ASCT should remain unchallenged and offered to all eligible patients, but with the presently available data, we believe it is a reasonable strategy to maximize PFS1. With the improved rates of MRD-negativity incorporating novel agents earlier in treatment, an important predictor of outcome and possible future surrogate endpoint for survival, we may be shifting to an era away from frontline ASCT and reserve it for progressive disease. Finally, while consolidation therapy post transplantation could induce deeper response rates, for the majority of patients, relapse remains inevitable, making the case for maintenance therapy with lenalidomide at the least until progression.
